# Role of Dexamethasone and Methylprednisolone Corticosteroids in Coronavirus Disease 2019 Hospitalized Patients: A Review

**DOI:** 10.3389/fmicb.2022.813358

**Published:** 2022-02-15

**Authors:** Jyoti Mehta, Rajan Rolta, Brij Bhushan Mehta, Neha Kaushik, Eun Ha Choi, Nagendra Kumar Kaushik

**Affiliations:** ^1^Faculty of Applied Sciences and Biotechnology, Shoolini University of Biotechnology and Management Sciences, Solan, India; ^2^Department of Immunology, University Institute of Oslo, Oslo, Norway; ^3^Department of Biotechnology, The University of Suwon, Hwaseong, South Korea; ^4^Department of Electrical and Biological Physics, Plasma Bioscience Research Center, Kwangwoon University, Seoul, South Korea

**Keywords:** COVID-19, dexamethasone, methylprednisolone, corticosteroids, inflammatory impacts, pharmacokinetics, pharmacodynamics

## Abstract

The WHO announced coronavirus disease 2019 (COVID-19) as a pandemic disease globally on March 11, 2020, after it emerged in China. The emergence of COVID-19 has lasted over a year, and despite promising vaccine reports that have been produced, we still have a long way to go until such remedies are accessible to everyone. The immunomodulatory strategy has been kept at the top priority for the research agenda for COVID-19. Corticosteroids have been used to modulate the immune response in a wide range of diseases for the last 70 years. These drugs have been shown to avoid and reduce inflammation in tissues and the bloodstream through non-genomic and genomic effects. Now, the use of corticosteroids increased the chance of survival and relief by combating the viral strong inflammatory impacts and has moved to the forefront in the management of patients seeking supplemental oxygen. The goal of this review is to illuminate dexamethasone and methylprednisolone, i.e., in terms of their chemical and physical properties, role in COVID-19 patients suffering from pneumonia, the proposed mode of action in COVID-19, pharmacokinetics, pharmacodynamics, clinical outcomes in immunocompromised populations with COVID-19, interaction with other drugs, and contradiction to explore the trends and perspectives for future research. Literature was searched from scientific databases such as Science Direct, Wiley, Springer, PubMed, and books for the preparation of this review. The RECOVERY trial, a massive, multidisciplinary, randomized, and open-label trial, is mainly accountable for recommendations over the usage of corticosteroids in COVID-19 patients. The corticosteroids such as dexamethasone and methylprednisolone in the form of medication have anti-inflammatory, analgesic, and anti-allergic characteristics, including the ability to inhibit the immune system. These drugs are also recommended for treating symptoms of multiple ailments such as rheumatic and autoimmune diseases, leukemia, multiple myeloma, and Hodgkin’s and non-Hodgkin’s lymphoma along with other drugs. Toxicology studies proved them safe usually at low dosage *via* oral or other routes.

## Introduction

Severe acute respiratory syndrome coronavirus 2 (SARS-CoV-2) is found to be the principal infectious agent of coronavirus disease 2019 (COVID-19) after it originated in China in December 2019 through a zoonotic vector (Zhu L. et al., 2020). Toward the end of December 2019, an occurrence of mysterious pneumonia was marked by several symptoms like fever, dry cough, fatigue, and occasional gastrointestinal manifestation in the wholesale market of Huanan seafood in Wuhan, China. Inflammatory organ damage can result among sick individuals with critical COVID-19 or patients having an extremely greater expression of inflammatory markers including ferritin, C-reactive protein (CRP), interleukin-1, and interleukin-6 (Huang et al., 2020; Moore and June, 2020). Inflammation is the host reaction to microbes or tissue damage. This issue arises when inflammation becomes chronic in rheumatoid arthritis, which results in a long-term accumulation of inflammatory cells in synovial joints and other diseases involving chronic bronchitis and colitis. Severe COVID-19 disease might be caused by an overreaction of the natural immunity that results in the enhanced inflammatory response that is commonly referred to as a cytokine storm. The resulting cytokine storm causes life-threatening inflammation in the lungs and also causes injury in severe conditions of COVID-19 patients.

Various therapeutic strategies have been found to reduce harm to an inflammatory organ in viral pneumonia, but glucocorticoids, a known “double-edged sword,” has been widely unsupported (Russell et al., 2020; Shang et al., 2020). Glucocorticoids act as an anti-inflammatory and immunomodulatory that leads to more secondary infections to the body or make the existing infection difficult to control and evolve into sepsis, septic shock, multi-organ failure, and finally death. Glucocorticoids have been found to cure disorders closely similar to COVID-19, SARS, Middle East respiratory syndrome (MERS), community-acquired pneumonia, and severe influenza (Siemieniuk et al., 2015; Arabi et al., 2018; Lansbury et al., 2020). There is the uncertainty of corticosteroids over their potential benefits or harms in severe influenza (Rodrigo et al., 2016), and even the meta-analysis of Lansbury et al. (2020) reported the association of corticosteroid treatment in influenza with increased mortality and hospital-acquired infection at high doses and low quality. Initiation of SARS-CoV-2 infection occurs with the viral adherence to angiotensin-converting enzyme 2 (ACE2) which is expressed on the cells all over the body especially in the lungs during type 2 pneumocyte. People with extreme and critical COVID-19 revealed an elevated level of inflammatory molecules such as interleukins (IL-2, IL-6, and IL-10), monocyte chemoattractant protein-1, interferon-γ inducible protein-10, granulocyte-macrophage colony-stimulating factor (GM-CSF), tumor necrosis factor-alpha, and lymphopenia (Huang et al., 2020; Liu et al., 2020; Wang W. et al., 2020). Corticosteroids have formerly been enough to treat respiratory diseases like critical obstructive pulmonary disease, severe bacterial pneumonia, and acute respiratory sickness. In China, glucocorticoids have also been preferred in severe conditions (Zhao et al., 2020), while various COVID-19 treatment protocols specified that glucocorticoids would be either contradicted or not suggested before the study was completed (Dagens et al., 2020). In adrenocortical deficiency conditions, natural glucocorticoids such as hydrocortisone and cortisone also have sodium-retaining features, so they are used as replacement therapy. Synthetic derivatives of these hormones, such as dexamethasone, are extensively recognized for their anti-inflammatory impacts on multi-organ systems. Dexamethasone does not contain the sodium-retaining characteristic of hydrocortisone and its derivatives at the same anti-inflammatory dosages. On October 30, 1958, the Food and Drug Administration [FDA^1^] has also approved dexamethasone use, but the scientific data on methylprednisolone, which is an intermediate-acting corticosteroid, are confined to date (Williams, 2018; WHO Rapid Evidence Appraisal for Covid-19 Therapies (REACT) Working Group, Sterne et al., 2020). Methylprednisolone is a glucocorticoid acquired from prednisolone having better potential than prednisone. On October 24, 1957, the Food and Drug Administration [FDA^1^] approved methylprednisolone; and recently, Zhu N. et al. (2020) observed that low-dose methylprednisolone was efficacious to avoid COVID-19-associated pneumonia for a patient having long-lasting immunodeficiency during the COVID-19 pandemic. Mechanistically, methylprednisolone has a higher lung tissue-to-plasma ratio in experimental animals in comparison with dexamethasone, which is more potent in lung injury (Annane et al., 2017). The corticosteroids such as dexamethasone and methylprednisolone are used in dealing with numerous diseases such as multiple myeloma, rheumatic disorders, respiratory diseases, renal diseases, ocular diseases, hematologic problems, neoplastic diseases, nervous disorder, gastrointestinal illness, endocrine dysfunction, and dermatologic problems. This review focuses on dexamethasone and methylprednisolone in terms of their chemical and physical properties, role in COVID-19 during pneumonia infection, proposed mode of functioning during COVID-19, and their pharmacokinetics, pharmacodynamics, interaction with other drugs, and contradiction.

## Materials and Methods

The present review collected the literature published on chemical and physical properties, role mode of action, application, administration, pharmacokinetics, pharmacodynamics, interaction with other drugs, safety aspects, and contradictions of both corticosteroids like dexamethasone and methylprednisolone *via* several scientific search engines including Google Scholar, Science Direct, Springer, PubMed, SciFinder, Wiley Online Library, and Tylor & Francis. The International Union of Pure and Applied Chemistry (IUPAC) names and chemical structures of drugs were obtained *via* PubChem database and ChemDraw Pro 8.0 software, respectively. Pictorial representation of pathological events including the corticosteroid role in COVID-19 infection was done by BioRender software.

## Chemical and Physical Characteristics of Dexamethasone and Methylprednisolone

### Dexamethasone

**FIGURE 1:**
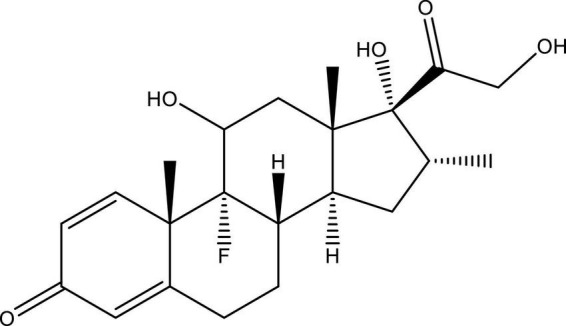
Structure of 9α-fluoro-16α-methylprednisolone (dexamethasone). This figure is made using ChemDraw.

Dexamethasone is a crystalline white powder that is odorless and water-insoluble but sparingly miscible in anhydrous ethanol and slightly shows miscibility in methylene chloride. When exposed to air, it remains stable (≤ 0.1 mg/ml) (U.S. National Library of Medicine). The molecular formula and molecular weight of dexamethasone are C_22_H_29_FO_5_ and 392.47 Da, respectively. It is chemically known by different names such as 9α-fluoro-16α-methylprednisolone, 9α-fluoro-llβ,17α,21-trihydroxy-16α-methylpregna-1,4-diene-3,2*O*-dione, or 16α-methyl-9α-fluoroprednisolone and is structurally illustrated in Figure 1.

### Methylprednisolone

**FIGURE 2:**
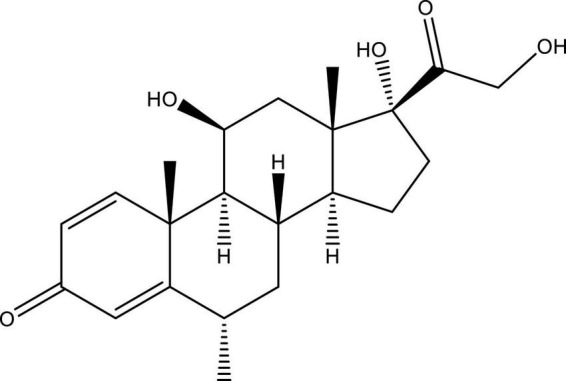
Structure of 11β,17,21-trihydroxy-6α-methylpregna-1,4-diene-3, 20-dione (methylprednisolone). This figure is made using ChemDraw.

Methylprednisolone acetate is a 6-methyl derivative of prednisolone that is an odorless and relatively white crystalline powder and melts with certain degradation at approximately 213°C. It is miscible in several solvents like dioxane, acetone, alcohol, chloroform, and methanol and shows slight dissolubility in ether and insolubility in water. The molecular formula of methylprednisolone is C_22_H_30_O_5_, and its molecular weight is 416.51 Da. Chemically, methylprednisolone acetate is known as 11β,17,21-trihydroxy-6α-methylpregna-1,4-diene-3,20-dione and pregna-1,4-diene-3,20-dione and is structurally represented in Figure 2.

## Role of Dexamethasone and Methylprednisolone During COVID-19 Infection

Corticosteroids are readily accessible, cheap, and easy to employ, so they were examined in different observational studies to explore their effectiveness in COVID-19 treatment (Fadel et al., 2020; Salvi and Patankar, 2020). However, during the initial 6 months of the pandemic, treatment practices varied greatly worldwide, but it was found from several reports that up to 50% of patients were restored with glucocorticoids (Wang D. et al., 2020; Xu et al., 2020). The literature evidence that supports or discourages glucocorticoids in these situations has been inadequate in the controlled trial studies (Lansbury et al., 2020). Glucocorticoids may also play role in reducing inflammation-mediated lung damage and therefore lessens the severity leading to respiratory discomfort and even death. A recent analysis mentioned that many patients died due to acute respiratory distress syndrome (ARDS) triggered by the cytokine storm, a fatal uncontrolled systemic inflammation developed due to the secretion of a huge amount of pro-inflammatory cytokines and chemokines from adaptive immune cells during a situation of SARS-CoV infection (Guo et al., 2020). Along with inconsistent data in several other viral types of pneumonia about the safety and positive benefits of corticosteroids, WHO announced early in the pandemic guidelines for using such drugs regularly for treating COVID-19 patients (Papamanoli et al., 2020). However, it is well known that when taken at the proper time during infection, glucocorticoids can help during the inflammatory storm by affecting pro-inflammatory gene expression and eventually decreasing cytokine levels (Darwish et al., 2011). Medical professionals, as well as frontline doctors, are currently targeting antiviral, antimalarial, and anti-inflammatory components, which have been around for several years and are widely accessible, for example, hydroxychloroquine, chloroquine, remdesivir, lopinavir, and corticosteroids. There are though just a few clinical trials that have employed corticosteroids to treat COVID-19 patients, and several findings carry many variations related to dose, corticosteroid type, duration of therapy, and which patients are suited for the therapy (Liu et al., 2020). The guideline panel of “The Infectious Diseases Society of America” (Clinical Practice Guidelines by the Infectious Diseases Society of America (IDSA) on the Treatment and Management of Patients with COVID-19, 2020) has recommended glucocorticoids in extreme COVID-19 patients (with SpO_2_ ≤ 94% on room air) who demand supplemental oxygen, extracorporeal life support, or assisted ventilation. A multicenter analysis done in China revealed that during COVID-19 severity, serum lactate dehydrogenase (LDH) levels become greater than twice the normal limit, which might be a favorable condition for initiating corticosteroid therapy with a low-to-medium dosage to promote viral clearance and reduce mechanical breathing necessity (Li et al., 2020).

It is mentioned on the WHO Model List of Essential Medicines since 1977 that dexamethasone is a fluorinated manmade adrenocortical steroid and is already off-patent and accessible in multiple countries. Dexamethasone is a widely accessible glucocorticoid-like drug used for allergies and pain that has emerged as a dark horse in the battle to identify remedies and thus save lives from the emerging coronavirus pandemic or COVID-19. The medicine is now being hailed as the first COVID-19 lifesaver. The preliminary clinical study performed in the United Kingdom suggested that dexamethasone could be able to enhance the survival of extremely ill COVID-19 patients. On June 16, 2020, scientists issued a press release on trial analysis with an announcement that dexamethasone, an inexpensive and readily accessible medicine, could lessen the fatality rate remarkably during the critical condition of COVID-19 patients (Oxford University Press Release, June 16). However, a respiratory infection desired hospitalization occurs in a large percentage of patients (Verity et al., 2020) and could also escalate to critical disorders with hypoxemic respiratory distress demanding prolonged ventilator support (Chen et al., 2020; Ruan et al., 2020). The RECOVERY trial recently revealed abrupt mortality declines while receiving dexamethasone initially during the therapy of COVID-19 hospitalized patients who desired oxygen or ventilators (RECOVERY Collaborative Group et al., 2021). These observations support and extend the existing outcomes from several newly reported randomized and cohort analyses from Italy, Spain, and the United States, which show interaction among corticosteroid prophylaxis, lower mortality, and a need for ventilation systems (Fadel et al., 2020; Salton et al., 2020; Corral-Gudino et al., 2021). According to preliminary reports, the medication (6 mg for 10 days) lowered death by 1/3 in patients on invasive mechanical ventilators and 1/5 in patients requiring only oxygen (RECOVERY Collaborative Group et al., 2021). The full data of the trial have not yet been released, although they are expected soon. The preliminary screening has sparked curiosity in the medication, which has been in use since the 1960s. The European Medicines Agency (EMA) endorsed the dexamethasone in adults and adolescents (age > 12), and having a weight **≥** 40 kg demands surplus oxygen therapy (European Medicines Agency [EMA], 2020). Dexamethasone or MK-125 has a significant role in different disorders related to endocrine, dermatologic, ophthalmic, allergic, gastrointestinal, rheumatic, hematologic, neoplastic, collagen, respiratory, edematous, and other conditions.

Dexamethasone has an anti-inflammatory potential that may help to mitigate an uncontrolled inflammatory response. Zydus Cadila is the largest manufacturer of dexamethasone in India. Dexamethasone is among the 53 medicines identified by the government of India as being eligible for the recently announced production-linked incentive program. The same data of the corticosteroids used against COVID-19 are now in line with frontline professionals’ perspectives in Wuhan (Shang et al., 2020), and the latest report is released from Spain (Fernández-Cruz et al., 2020). An *in vitro* analysis revealed that a 0.1-μM concentration of dexamethasone added to human alveolar epithelial cells including H441 and A549 could result in the suppression of cell growth and shortening of the recovery period as compared with the normal cell (Nalayanda et al., 2014). Furthermore, previous research has mentioned that low dosages of dexamethasone (0.4 mg/kg/day in 4 doses after 48 h) were effective in respiratory syncytial virus (RSV)-infected patients, causing lung infections (Van Woensel et al., 2003). A dose of dexamethasone given at 0.6 mg/kg/day to patients having bronchiolitis caused by RSV was approved for having positive benefits. Understanding the necessity of gathering accurate reports on the potency of corticosteroids to improve clinical management, WHO approved a strategy for a prospective meta-analysis (Thomas et al., 2021) of ongoing randomized clinical trials. A prospective meta-analysis of these including other trials, involving 1,703 individuals in whom 1,007 participants (59%) were from the RECOVERY trial confirmed a reduction in mortality within 28 days with little heterogeneity across investigations (WHO Report, 2020). Dexamethasone was proven to be helpful only in extremely ill COVID-19 patients, according to the RECOVERY trials. The latest prospective meta-analysis explored seven clinical trials (CAPE COVID, DEXA-COVID 19, COVID STEROID, CoDEX, RECOVERY, REMAP-CAP, and Steroids-SARI) across 5 continents to determine the potency of corticosteroids in an extreme condition of COVID patients (WHO Report, 2020).

**FIGURE 3:**
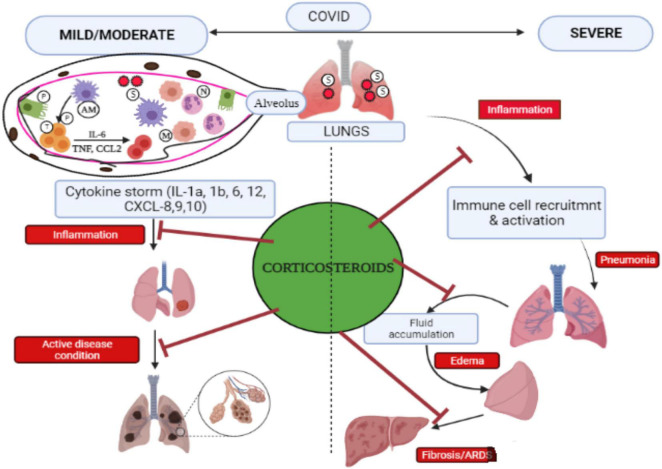
Pictorial representation of pathological events that occurred during COVID-19 and the efficacy of corticosteroid to control COVID-19 infection. SARS-CoV-2 gets inside the host *via* the pulmonary route, penetrates the alveoli, and interacts with alveolar macrophages (AMs) and pneumocytes (P). Infectious AMs release cytokines/chemokines that recruit and trigger other immune cells such as T cells (T) to produce cytokines, causing “cytokine storm,” and monocytes (M) along with neutrophils (N) move from the blood to the infection site. The lungs in COVID-19 cases in severe condition at late stage lead to acute respiratory distress syndrome (ARDS) and pulmonary fibrosis. Corticosteroids help in inhibiting inflammation caused by cytokine storms and prevent several problems associated with severe COVID-19 including pneumonia, edema, ARDS, and fibrosis. Adapted and modified from Gopalaswamy and Subbian (2021) with permission.

Moreover, in initial COVID-19 analyses, changes in corticosteroid doses and administration led to inconsistent data regarding the efficiency of such drugs (Stockman et al., 2006). Later research studies, however, have shown that methylprednisolone is efficacious in COVID-19 patients and hospitalized hypoxic COVID-19 individuals (Papamanoli et al., 2020; Ranjbar et al., 2021), while Fatima et al. (2020) detected that both dexamethasone and methylprednisolone are equally efficient in managing COVID-19 illness from mild to extreme conditions. For individuals with ARDS provoked by SARS-CoV-2, Hazbun et al. (2020) experimented with methylprednisolone (125 mg/6 h for 24 h, thereafter 60 mg/12 h for 10 days). In this finding, the alveolar-arterial (A-a) O_2_ gradient was lowered to 228 mmHg from 455 mmHg within 2 days, indicating improved oxygen diffusion from the lungs to the bloodstream. Furthermore, following 8 days of treatment, 95% were relieved from mechanical ventilation. Unfortunately, the whole sample of patients developed hyperglycemia after receiving methylprednisolone, which was due to insulin (Wu et al., 2020). Another finding proved that giving a standard dose of methylprednisolone during pneumonia caused by coronavirus could considerably decrease mortality risk by 62% (Wu et al., 2020). When the patients with COVID-19 do not feel any respiratory trouble, researchers include a lesser number of participants and found no link of methylprednisolone dosage with clinical outcome (Ding et al., 2020; Zha et al., 2020). One of the studies conducted in the Henry Ford Health System (HFHS)-centralized clinical microbiology laboratory detected that methylprednisolone for a shorter time duration (0.5–1 mg/kg/day in two divided doses for 3 days) could lessen the necessity for escalation care and promote clinical outcomes in COVID-19 patients affected with mild-to-severe conditions (Fadel et al., 2020). Pathological events that occurred in COVID-19 and corticosteroids are seen to be successful in suppressing COVID-19 infections in both severe and mild symptoms (Figure 3). The positive impacts of corticosteroids for chronically ill COVID-19 hospitalized individuals have been proved, but there are still a few unanswered questions and problems that require to be discussed and addressed in future work.

## Dexamethasone and Methylprednisolone in COVID-19 Pneumonia Patients

Prophylaxis for SARS-CoV-2 pneumonia patients included a plethora of antibiotics, antivirals, and, in certain instances, corticosteroids at different concentrations. The use of corticosteroids seemed to be controversial during the initial phase of the COVID-19 outbreak. In a placebo-controlled, randomized, multicenter analysis of Villar et al. (2020), dexamethasone proved to be beneficial in patients with ARDS by minimizing the risk of death from 36 to 21% with a *p*-value < 0.0047. Wu et al. (2020) conducted a study on ARDS patients induced by COVID-19 who were found to be cured with methylprednisolone and had a little mortality risk (hazard ratio (HR), 0.38; 95% CI, 0.20–0.72). The RECOVERY data have now changed the clinical guidelines (RECOVERY Collaborative Group et al., 2021); participants receiving dexamethasone had shown a decrease in the mortality rate in one-third of patients on ventilation and one-fifth of patients receiving only oxygen, although the variation in the mortality rate among those who received dexamethasone measuring 6 mg and those who did not take it was 22.9 and 25.7%, respectively. The higher number of deaths in COVID-19 individuals can be stated by the rapid development of Organized Pneumonia secondary to the SARS-CoV-2 virus, which has already been recorded in autopsies even during the first week of infestation. This disease usually requires the use of larger doses of corticosteroids, referred to as “pulse” doses for a prolonged period. As an outcome, the dose recommended *via* the RECOVERY trial may be insufficient for various patients (Pierre and Jeffrey, 2020). On June 16, 2020, the trial done on a preliminary basis had revealed the potential of dexamethasone at 6 mg given every day for 10 days (or until hospital discharge) during pneumonia in COVID-19 in decreasing 11% of the mortality rate, while those patients on invasive mechanical ventilation show mortality rate reduction from 40 to 29% (RECOVERY Collaborative Group et al., 2021). Yang R. et al. (2020) detected 175 people with chronic COVID-19 and prescribed them methylprednisolone to prove its role in preventing the progression of critical disease in the multivariate study (*p*-value < 0.001; odds ratio (OR): 0.054; 95% CI: 0.017–0.173). Edalatifard et al. (2020) compared 34 randomized people prescribed 250 mg/day of methylprednisolone for 3 days with an equal population receiving only standard care during COVID-19 pneumonia. They observed that clinical improvement was 94.1 and 57.1% in the methylprednisolone and standard care groups, respectively; and the mortality rate was only 5.9 and 42.9% in the methylprednisolone group and standard care group, respectively.

Ruiz-Irastorza et al. (2020) observed an elevated inflammatory marker in 242 patients having COVID-19 pneumonia and treated them with methylprednisolone pulses given at 125–250 mg/day for 3 days on the second week. They mentioned that HRs for death and incubation or death for patients were 0.35 (95% CI*:* 0.11–1.06, *p*-value: 0.064) and 0.33 (95% CI: 0.13–0.84, *p*-value: 0.020), respectively, and found methylprednisolone pulses efficient in improving the prognosis of patients suffering from COVID-19 pneumonia. HR with 95% CIs determines the strength of the linkage between risk predictors and results. Some studies suggested that corticosteroids have also been administered in pneumonia caused by COVID-19 (Wang Y. et al., 2020). Despite getting large doses of corticosteroids, there is no increased risk of superinfection, which could be associated with a short period of administration (Pinzón et al., 2021). One of the investigations compares population according to corticosteroid intake in which 111 patients were given dexamethasone and 105 patients received methylprednisolone. The dexamethasone group shows that critical ARDS in 26.1% of patients and 17.1% in the methylprednisolone group after 4 days decreased, with the laboratory severity markers like CRP, D-dimer, LDH, and even intensive care unit (ICU) reduced to 4.8% from 14.4% and mortality reduced from 17.1 to 9.5% in the methylprednisolone group. Therefore, it was concluded that recovery time was lesser among patients in the methylprednisolone high dose group (3–4 days) followed by prednisone given orally for 14 days, while the dexamethasone group includes 6 mg for 7–10 days (*p*-value < 0.0001). Therefore, after 30 days, 92.6% were alive in the methylprednisolone group but 63.1% in the dexamethasone group (Pinzón et al., 2021).

## Proposed Mechanism of Action of Corticosteroids in COVID-19

Corticosteroids have pleiotropic effects produced from complex molecular mechanisms involving non-genomic and genomic effects (Cain and Cidlowski, 2017). Dexamethasone binds to intracellular glucocorticoid receptors (GRs), which depend on ligand and transcription factors expressed by body cells along with naturally produced cortisol including synthetic counterparts. Ligand-linked GRs move from the cytoplasm to the nucleus and bind with particular DNA sequences located at the regulatory site of target genes leading to chromatin remodeling (Luecke and Yamamoto, 2005). Direct adherence to response sites of glucocorticoid located in target genes has genomic impacts, positive or negative transcription, and its attachment to other transcription factors *via* protein-protein interaction and affects gene transcription either by enhancing or through inhibition. Therefore, associations with plasma membranes, interfering in cytoplasmic signaling pathways, and mitochondrial translocation were among the mechanisms in which GRs can quickly impair non-genomic cellular activity.

**FIGURE 4:**
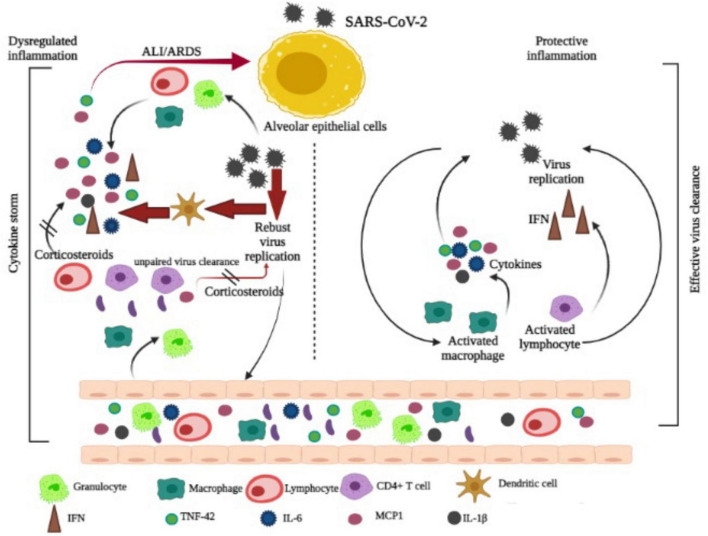
Mechanism of COVID-19 and corticosteroid action is adapted and modified from Yang Yang J.W. et al. (2020).

COVID-19 patients undergo a hyperinflammatory state during which a cytokine storm has similar characters with a rare hematological syndrome known as hemophagocytic lymphohistiocytosis in pregnant and postpartum women (Obuchowska et al., 2021). The immunosuppressive characteristics of dexamethasone are considered to be the reason for its effectiveness in COVID-19 victims. Glucocorticoids act as a modulator of multiple biological activities in immune cells or other organs and tissues of the human body, as they inhibit the adaptive immune defense system (Strehl et al., 2019). According to recent data, glucocorticoids may provide both stimulation and inhibition of the immune reaction depending on their dosage in the blood and the duration of time that drugs are administered (Singh et al., 2020). Immune suppressions during the initial viral infection may permit faster viral multiplication and worsen over time. The 3C-like proteinase presented over SARS-CoV-2 (nsp5) may block histone deacetylase 2 (HDAC2) from entering the nucleus and therefore limit its ability to control inflammation and cytokine responses; dexamethasone-induced histone deacetylase secretion may directly help in counteracting SARS-CoV-2. During severe COVID-19, corticosteroid therapy reduced the lengths of hospitalization (Wang Y. et al., 2020) and ICU stay, and treatment with methylprednisolone decreased the risk of death for individuals with COVID-19 with ARDS. A summarized potential mechanism of COVID-19 along with the action of corticosteroid is represented in Figure 4.

**FIGURE 5:**
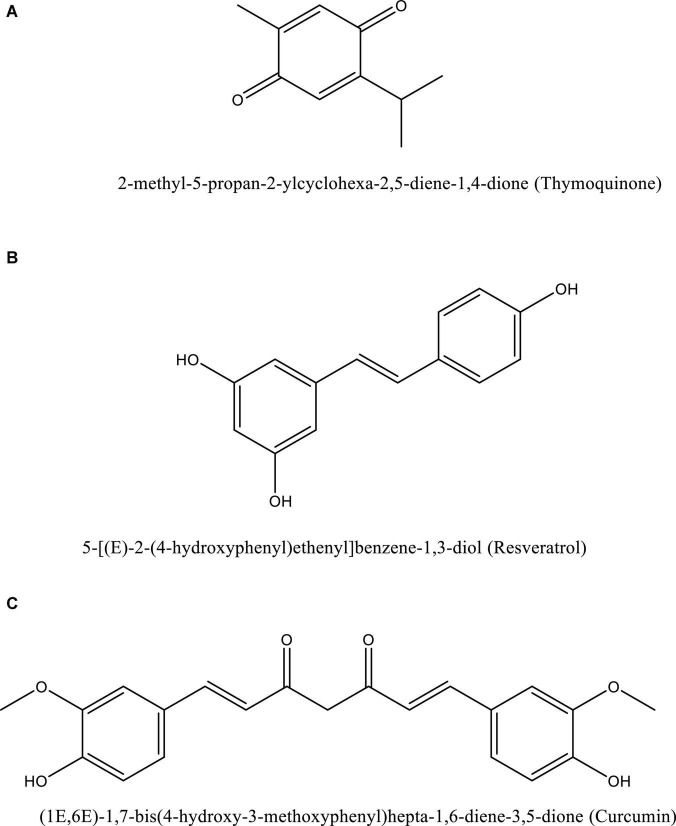
Structure of naturally occurring compounds such as thymoquinone **(A)**, resveratrol **(B)**, and curcumin **(C)**. These structures are prepared using ChemDraw software.

Dexamethasone decreases hemolysis and promotes heme oxygenase (HO)-1 in macrophages, resulting in a reduced severity among COVID-19 patients (Vallelian et al., 2010). Thymoquinone, resveratrol, and curcumin (Figure 5) are a few examples of natural compounds that can upregulate HO-1 (Funes et al., 2020).

Therefore, the phytocompounds extracted from medicinal plants could also be used as an alternative to synthetic drugs; even we have tried the same in our previous reports by using *in vitro* and *in silico* methods as shown in Table 1.

**TABLE 1:** Medicinal plants reported having antimicrobial activity including anti-SARS-CoV-2 activity.

S. no.	Medicinal plants	Pathogens	References
1	*Zingiber officinale*, *Allium sativum*, *Syzygium aromaticum*, *Berberis aristata*, *Rhus cotinus*, and *Phyllanthus emblica*	*Salmonella* species	Mehta et al., 2016, Mehta et al., 2021a,b
2	*Heracleum lanatum*, *Girardinia diversifolia*, *Chenopodium album*, *Solena amplexicaulis*, *Rheum austral*, *Rubus ellipticus*, *Verbascum Thapsus*, and *Viola canesiens*	*Staphylococcus* species	Urmila et al., 2016
3.	*Rheum emodi*, *Thymus serpyllum*, *Cymbopogon citrates*, *Moringa oleifera*, *Thalictrum foliolosum*, *Berberis aristata*, *Piper nigrum*, *Allium sativum*, *Myristica fragrans*, and *Zanthoxylum armatum*	SARS-CoV-2	Rolta et al., 2020

## Practical Application in Licensed Indications

### Dexamethasone

Dexamethasone is available in 35 different forms, according to the Electronic Medicines Compendium such as tablets and oral solutions, injection solutions, an ointment, and ear drops/spray and eye drops requiring 17 formulations, 6 formulations, 1 formulation, and 10 formulations, respectively, and also an intravitreal implant. The formulations range from 0.5 to 4 mg/tablet and from 2 to 20 mg/5 ml for oral solution. It is also assumed that dexamethasone at 2-mg tablets in various disorders is amenable to glucocorticoid remedy and as an adjuvant for cerebral edema. The solution for injection is indicated “For use in certain endocrine and non-endocrine syndromes susceptible to corticosteroid therapy.” Therefore, glucocorticoids have numerous therapeutic applications, and their practical implementation alters accordingly. For example, 1 mg of dexamethasone through the oral route can quench adrenal gland-released cortisol (hydrocortisone) in healthy individuals examined *via* dexamethasone suppression test. A daily dose of 16 mg in brain tumors could relieve elevated intracranial pressure. In adults, oral dosage levels of 0.5–10 mg/day have been used to treat inflammatory and allergic reactions.

### Methylprednisolone

Methylprednisolone suppresses cell-mediated immunologic responses, notably those that relied on lymphocytes. The methylprednisolone–GR complex inhibits promoter domains of pro-inflammatory genes, leads to the improvement of anti-inflammatory gene product expression (Scheinman et al., 1995), and hinders the secretion of inflammatory cytokines, chiefly by retarding the nuclear factor-kappa-B (NF-κB) (Jaime-Pérez et al., 2013). Edalatifard et al. (2020) carried a randomized trial to determine the efficacy of methylprednisolone pulse given intravenously and thereby confirmed that intake of methylprednisolone had reduced the death rate and extended the survival period. *In vitro* screening indicated that it helps in reducing CRP levels while increasing platelet count. Contrary to this report, another study stated that standard care with dexamethasone given confirmed the superiority of methylprednisolone over dexamethasone dosage alone. Wang Y. et al. (2020) assessed the action of low dosages of methylprednisolone for a shorter-term span and observed that patients taking 1–2 mg/kg/day for 5–7 days seemed to have reduced hospital course span and less need for respiratory support but still similar in death rate compared with those receiving standard care in COVID-19. Further research has shown that people using methylprednisolone have minimal negative consequences (Corral-Gudino et al., 2021; Jeronimo et al., 2021; Nelson et al., 2021). One key goal during COVID-19 is to lessen inflammatory reaction while using anti-inflammatory agents such as dexamethasone, prednisone, methylprednisolone, and hydrocortisone. Dexamethasone is recommended to be taken at a daily 6-mg dose either orally or intravenously for resolving serious COVID-19 conditions. If dexamethasone is not accessible, the US National Institutes of Health suggests utilizing alternate corticosteroids at equal dosages like prednisone at 40 mg, methylprednisolone at 32 mg, and hydrocortisone at 160 mg. When using alternative corticosteroids, the half-life, time of activity, and supplementation frequency should all be thoroughly examined.^2^ Several scholars revealed that methylprednisolone has higher penetration inside the lungs especially in contrast to dexamethasone which was reported to have better outcomes (Hirano et al., 1998). The observed differences suggest that 6 mg of dexamethasone per day would be as effective as 32 mg of methylprednisolone (Mager et al., 2003). Methylprednisolone surpassed dexamethasone among hypoxic COVID-19 patients who have been hospitalized (Ranjbar et al., 2021).

## Indications and Usage

### Oral Route Administration

#### The Allergic States

Dexamethasone is used to control intense or aggravating allergic conditions seen in various disorders like asthma, eczema, contact dermatitis, allergic rhinitis, drug hypersensitivity reactions, and even serum sickness. The National Asthma Education and Prevention Program (NAEPP) recommends methylprednisolone in long as well as short-term medications to manage and inhibit persistent asthma (National Asthma Education and Prevention Program [NAEPP], 2007). During aggravation, methylprednisolone given orally is recommended over the intravenous route except in troubles related to adherence or vomiting.

#### Dermatologic Diseases

Dexamethasone was firstly used to treat extensive, chronic, and acute skin problems such as erythroderma, vitiligo vulgaris, and extreme erythema multiforme (Stevens–Johnson syndrome). Methylprednisolone helps to combat facial and scalp eczema, as well as sunburn, and has exhibited significant efficacy in psoriasis treatment. Methylprednisolone is especially ideal for usage in children and infants owing to its faster efficacy with a lack of adverse local and/or systemic effects (Ruzicka, 2006). Methylprednisolone acetate excelled as tacrolimus ointment (0.03%) in terms of Eczema Area and Severity Index (EASI), itching, and sleep disturbance (Bieber et al., 2007).

#### Endocrine Disorders

Hydrocortisone or cortisone is prescribed in primary or secondary adrenocortical insufficiency, which may also be used in addition to synthetic mineralocorticoid analogs in several conditions like congenital adrenal hyperplasia, hypercalcemia associated with cancer, and non-suppurative thyroiditis. However, Eishi et al. (1983) did intrauterine access to the fetus with dexamethasone, which proved to be a potential modulator of the development and performance of endocrine and immunological organs, resulting in premature death or growth retardation after birth. In the same finding, methylprednisolone is used as a second-line treatment when combined with mineralocorticoids in primary or secondary adrenocortical insufficiency.

#### Gastrointestinal Diseases

Corticosteroids proceed to be the central part of anti-inflammatory and immunosuppressive treatment for a diverse variety of disorders. Corticosteroids remain one of the effective therapies in the medication of inflammatory bowel disease (IBD) (Speiser et al., 2010), regional enteritis, and ulcerative colitis.

#### Hematologic Disorders

Dexamethasone is used for autoimmune diseases, hemolytic anemia, erythroid hypoplastic anemia, idiopathic thrombocytopenic purpura in adults, secondary thrombocytopenia, and erythroblastopenia. Similarly, methylprednisolone also serves to treat various disorders such as autoimmune hemolytic anemia, congenital (erythroid) aplastic anemia, and immune thrombocytopenia (Jaime-Pérez et al., 2013).

#### Neoplastic Diseases

Both dexamethasone and methylprednisolone are used in leukemia and lymphoma management.

#### Nervous System Disorder

Dexamethasone is used in acute exacerbations of multiple sclerosis, neuro-surgical intervention, craniotomy, and cerebral edema seen in a primary or metastatic brain tumor and head injury, while methylprednisolone-containing therapy is useful in intense exacerbations of multiple sclerosis (Murray, 2006).

#### Ophthalmic Diseases

Dexamethasone is used for sympathetic ophthalmia, ocular inflammatory, temporal arteritis, and uveitis conditions, while methylprednisolone is a therapeutic choice during severe allergy and inflammation in the eye and its adnexa causing uveitis, scleritis, chorioretinitis, iritis, and iridocyclitis, keratitis, optic neuritis, retinal vasculitis, and allergic conjunctivitis.

#### Renal Dysfunction

Dexamethasone is given to remit proteinuria in idiopathic nephrotic syndrome or lupus erythematosus and treat diuresis, while methylprednisolone is helpful in treating nephrotic syndrome, idiopathy, or lupus nephritis (Illei et al., 2001; Charkoudian et al., 2012).

#### Respiratory Diseases

Dexamethasone might be able to exert chronic asthma exacerbations when used orally. Methylprednisolone is responsible for curing several conditions related to pneumonitis, asthma, and chronic beryllium disease as a supplement to antituberculosis chemotherapy during disseminated pulmonary tuberculosis, eosinophilic pneumonia, and symptomatic sarcoidosis (Alangari, 2014; Zhao et al., 2016).

#### Rheumatic Disorders

Both dexamethasone and methylprednisolone are used as supplemental therapy for a short duration to tide the patient over an acute or exacerbated acute gouty arthritis, acute rheumatic carditis, Bekhterev’s disease, psoriatic arthritis, rheumatism, including juvenile idiopathic arthritis (Badsha and Edwards, 2003; Nash, 2006), polymyositis, and systemic lupus erythematosus. This is most typically injected into the knee and shoulder joints (Habib, 2009).

#### Multiple Myeloma

Daratumumab and hyaluronidase-fihj (Darzalex Faspro is an anti-CD38 monoclonal antibody) have been approved by the FDA when combined with pomalidomide and dexamethasone in treating multiple myeloma that had relapsed for the first or second time (Janssen, 2021). Multiple myeloma is leukemia that affects plasma cells and seems to be incurable. When these cells are injured, they spread quickly and replace normal cells in the blood marrow with tumors. As per the press release, an estimated 34,000 people in the United States were diagnosed with multiple myeloma in 2021, with close to 12,500 dying from the condition. When comparing pomalidomide and dexamethasone alone, the research met its primary goal of improved progression-free survival (PFS), with a substantial 37% lower risk of progression or death. Dexamethasone has also been used in veterinary medicine to treat an array of challenges in farm and companion animals, including inflammation, acetonemia, skin disease, stress, and shock.

### Parenteral Route

#### Soft Tissue or Intra-Articular Injections

Intra-articular corticosteroid injections are an integral part of rheumatologic practice in the management of numerous articular and peri-articular inflammation and pain to provide local, immediate anti-inflammatory results in both adults and children and as an adjuvant to systemic drugs (Chan, 2015). Intra-articular corticosteroids are suggested in treating different conditions including acute gouty arthritis, severe and mild tendonitis, epicondylitis, and synovitis of osteoarthritis (Garg et al., 2014). Intra-articular routes for corticosteroids are a second-line option for rheumatoid arthritis-related joint discomfort (Habib, 2009).

#### Intralesional Injections

Intralesional corticosteroid injections are used to treat several dermatological and non-dermatological disorders with variable results. The indications for intralesional corticosteroid therapy are acute and chronic, inflammatory processes (Richards, 2010), hyperplastic and hypertrophic skin disorders such as vitiligo capitis, discoid lupus erythematosus, necrose, granuloma annulare, lichen ruber planus, lichen simplex chronicus, psoriatic plaques, and necrobiosis lipoidica diabeticorum (Syed et al., 2013; Senila et al., 2015).

#### Intramuscular Injections

Methylprednisolone and dexamethasone help to treat many of the same issues as oral medications. As an option for oral medication, intramuscular injections are used. Intramuscular injection sites include the upper outer thigh area muscle, shoulder muscle, upper arm, and also hip. Intramuscular injections are used for the treatment of trigger points related to myofascial pain syndrome. Intramuscular injections of corticosteroids appear to be as effective as corticosteroid tablets in preventing relapse (Kirkland et al., 2018).

## Pharmacokinetics of Methylprednisolone and Dexamethasone

Dexamethasone is readily absorbed if taken orally, and the highest plasma levels are acquired within 2 h after ingesting and vary considerably between individuals. The average plasma half-life is 3.6 ± 0.9 h. Dexamethasone is highly bound (approximately 77%) to albumin, which is a plasma protein. Dexamethasone indicates dose-independent pharmacokinetics in healthy volunteers (Rohdewald et al., 1987). Peterson (1959) reported the first pharmacokinetic analysis, revealing a half-life of 3.3 h in normal individuals employing infusions of “pharmacological” doses of dexamethasone. After injecting 1.5 mg of dexamethasone intravenously, Haque et al. (1972) determined dexamethasone mean clearance (201 ml/min), the volume of distribution (43.6 L), and half-life (4.2 h). One group of 15 patients experiencing community-acquired pneumonia received 6 mg of dexamethasone orally, whereas the other received 4 mg of dexamethasone intravenously in an assessment of dexamethasone kinetics. Although both groups had an apparent volume of distribution of 1 L/kg, the half-lives following oral and intravenous injection were approximately 7 and 9 h, respectively. However, dexamethasone has 36–54 h of biological half-life, while prednisolone has 18–36 h. It is approved by the WHO for the treatment for intramuscular administration of dexamethasone phosphate in 4 doses each of 6 mg/12 h. The dose dependency of dexamethasone pharmacokinetics and its effect on endogenous cortisol secretion were seen in healthy females. The highest plasma level occurred within 1.6–2 h after doses of 0.5–3.0 mg, confirming its independence on the administration route. Meikle et al. (1973) showed that dexamethasone given at 1 mg orally could indicate peak plasma limit between 30 and 60 min and stated that plasma dexamethasone levels given either orally or intravenously may be measured by radioimmunoassay. A similar observation was shown after intravenous injection of dexamethasone at 2 mg/kg (Hichens and Hogans, 1974). English et al. (1975) reported the dexamethasone disposition given orally at 2 mg in healthy volunteers and that peak plasma levels of dexamethasone remain within 8.5 and 27.0 ng/ml (23–72 nmol/L). Duggan et al. (1975) compared the bioavailability of dexamethasone drug (78.0 ± 12.1%) and elixir (82.6 ± 17.7%) to that of an intravenous infusion. In the same year, Hare et al. (1975) suggested that after providing dexamethasone phosphate through intravenous infusion, about 90% was converted to dexamethasone *in vivo*. In 1983, Brophy et al. (1983) stated that intravenous drug clearance was relatively significant (nearly 65% of expected hepatic plasma flow), and even higher oral clearance but constant absorption rate indicated its quick absorption after being given orally. Dexamethasone prescription is attributable to the drug’s higher clearance levels in individuals with neurological impairment than in active volunteers after a single dose.

There have been very few reports on the pharmacokinetics of dexamethasone. Researchers also confirmed that clearances of 200–250 ml/min and half-life of 2.5–4.5 h were observed, with a volume of distribution ranging from 40 to 60 L, indicating its increase with body size. Dexamethasone is currently used at 6 mg/day for patients with COVID-19 (Cain and Cidlowski, 2020). Both dexamethasone and its enantiomer, betamethasone, adhere to plasma proteins moderately (approximately 65%) (Ke and Milad, 2019). It is commonly given as the sodium phosphate ester/salt and hydrolyzed phosphate ester formed within 1 h (Rohdewald et al., 1987). Dexamethasone is partly metabolized by hepatic enzymes (Gentile et al., 1996), and its clearance after 4 doses is 0.18 L/h/kg and about 1.1 L/kg of steady-state volume in healthy males (Rose et al., 1981).

Jobe et al. (2020) observed pharmacokinetics of single 6-mg doses of dexamethasone phosphate and betamethasone phosphate alone, or an equal concoction of betamethasone phosphate with betamethasone acetate given orally and intramuscularly in reproductive-aged South Asian women. The terminal half-life of intramuscular or oral betamethasone is 11 h, which is approximately twice that of the mixture of oral and intramuscular dexamethasone (about 5.5 h). WHO approved maternal intramuscular therapeutics with 4 doses of dexamethasone phosphate at 6 mg/12 h, or 2 doses of beta phosphate and beta acetate at 12 mg/24 h at equal proportion (World Health Organization [WHO], 2015). The conventional treatment for women at risk of early delivery is high-dose prenatal corticosteroids (Roberts et al., 2017). Women who are at risk of preterm delivery were recommended to intake betamethasone and dexamethasone to avoid respiratory distress syndrome and death. Dosage of dexamethasone Krka at 4, 8, 20, and 40 mg have anti-inflammatory and anti-allergic effects. According to Krzyzanski et al. (2021), dexamethasone taken either orally or intramuscularly could produce very similar results in patients with COVID-19; but contrary to this report, Elliott et al. (1996) showed that the peak plasma concentration was 65% through the intramuscular route and the oral dosage bioavailability was 72% that of the intramuscular route of dexamethasone. To achieve identical maternal plasma pharmacokinetics to the current intramuscular dosage, a comparable high concentration of oral dexamethasone is recommended. The pharmacokinetic features of orally and intramuscularly given dexamethasone and betamethasone might be employed in designing new treatment strategies to reduce drug exposure. In high-resource settings, drug coverage is about 90% among women at risk of premature delivery (Jobe and Goldenberg, 2018). However, drug development is quite inadequate in numerous low-resource locations with the least maternal and newborn care, partly due to dubious efficacy and limited drug availability (Glasziou et al., 2017).

The bioavailability of methylprednisolone is 89.9% from the gastrointestinal tract when given *via* the oral route (Garg et al., 1979). Unlike endogenous glucocorticoids, methylprednisolone does not exhibit any binding to the glycoprotein transcortin (corticosteroid-binding globulin) but binds to albumin moderately. Therefore, the pharmacokinetics of methylprednisolone stays linear and does not rely on dose (Czock et al., 2005). Individuals with low albumin contents are more likely to encounter complications after glucocorticoid prescription. At 1.38 L/kg, oral methylprednisolone has a mild tissue distribution. Methylprednisolone is effectively excreted *via* hepatic metabolism and extrusion of metabolites, with a renal clearance of unaffected methylprednisolone for only 1.3–9.2% (Szefler et al., 1986). Methylprednisolone and methylprednisolone can be interconvertible. One dosage of dexamethasone daily might improve adherence. A dosage of 6 mg dexamethasone is equally effective (in terms of glucocorticoid activity) to 150 mg of hydrocortisone (50 mg given after every 8 h), 40 mg of prednisone, or 32 mg of methylprednisolone (e.g., 8 mg per 6 h or 16 mg per 12 h). Hence, it is advisable to keep checking sugar levels in chronic and critical conditions of COVID-19 patients, irrespective of whether the individual is reported to have diabetes. 11β-Hydroxysteroid dehydrogenases and 20-ketosteroid reductases regulate metabolism in the liver. Methylprednisolone expels hydrophilic inactive byproducts in the kidney, namely, 20-carboxymethylprednisolone and 6[β]-hydroxy-20[α]-hydroxy methylprednisolone (Czock et al., 2005). An open-label random report mentioned that methylprednisolone had no observable effect on the primary endpoint in the intention-to-treat study; however, another analysis revealed its advantages in COVID-19 patients (Corral-Gudino et al., 2021), which is similar to other published trials supporting glucocorticoid use in critical COVID-19 people demanding mechanical ventilation at the time of randomization in the United Kingdom (RECOVERY Collaborative Group et al., 2021). Efficacy was also observed in patients if the drug is administered for more than 7 days after the symptom appearance, where lung inflammation is more frequent. A recent meta-analysis of 7 glucocorticoid trials for severely sick individuals with COVID-19, comprising RECOVERY, supported the trial’s findings (WHO Rapid Evidence Appraisal for Covid-19 Therapies (REACT) Working Group, Sterne et al., 2020).

The WHO clinical guidance for COVID-19 defined the severity of COVID-19 by clinical indicators by reducing the oxygen saturation threshold from 94 to 90% (World Health Organization [WHO], 2020). Table 2 is adapted from WHO COVID-19 disease severity categorization.

**TABLE 2:** Different categories of disease severity during COVID-19 according to WHO guidelines.

COVID-19 condition	Definition
Critical Severe Non-severe	Acute respiratory distress syndrome (ARDS), septic infection, and even shock. It also involves other conditions that would normally require life-sustaining therapies like invasive/non-invasive mechanical ventilation or vasopressor therapy. 1. Oxygen saturation is less than 90% on room air. 2. Respiratory rate is greater than 30 breaths/min in adults and also in children of more than 5 years old, ≥ 60 breaths/min in infants of less than 2 months old, breaths/min ≥ 50 in infants of 2–11 months old, and breaths/min ≥ 40 in children of 1–5 years old 3. Respiratory distress includes the use of accessory muscle, inability to complete whole sentences; and in children, very severe chest walls indrawing, grumble, cyanosis, or other dangerous signs No signs of severe or critical conditions of COVID-19

## Pharmacodynamics of Methylprednisolone and Dexamethasone

Dexamethasone is a long-acting glucocorticoid having no retention features for sodium. It is mostly exploited as an anti-inflammatory and immunosuppressive drug. Its underlying mechanism is obtained *via* GR upregulation resulting in increasing or reducing the genes of transcription, which are important in chronic inflammation stimulating the inhibition of cytokine gene transcription and even making a direct link of the GR with other transcription factors. Dexamethasone has a biological half-life of 36–54 h, which is ideal for situations demanding persistent glucocorticoid activity.

Methylprednisolone is another glucocorticoid that exerts multiple phenotypic expressions by several physiological mechanisms (Timmermans et al., 2019). However, it is recommended extensively for inflammation and immunity (Xavier et al., 2016). Methylprednisolone is highly dependent on its linkage with intracellular GRs in contrast to being less dependent on mineralocorticoid receptors (MRs) that revealed a restricted distribution of tissue (Paragliola et al., 2017). Hence, the ligand-bound GRs transported to the nucleus help in changing the gene expression (Fernandes et al., 2019) and could have a wide array of adverse consequences ranging from hours to days. Glucocorticoids play an important function *via* limiting neutrophil death and demargination and reducing phospholipase A2, which suppresses the arachidonic acid lineage so they inhibit NF-κB and other inflammatory transcription factors but enhance the genes required for anti-inflammation including interleukin-10.^3^

## Clinical Outcomes of the Two Drugs in Immunocompromised Populations With COVID-19

A clinical trial of methylprednisolone and dexamethasone in immunocompromised populations could be completed in home-quarantined patients with COVID-19 when a low dose of methylprednisolone is given orally and dexamethasone was given intravenously in the first interval.^4^

## Drug–Drug Interactions

Generally, many possibly lethal drug–drug interactions have been recognized with corticosteroids and with dexamethasone particularly. When added to non-steroidal anti-inflammatory drugs (NSAIDs) or nicorandil, dexamethasone promotes the risk of peptic ulcers and bleeding, as well as gastrointestinal ulceration. Therefore, NSAID or nicorandil is not prescribed for individuals suffering from severe COVID-19. Dexamethasone can lower the level of potassium, which enhances the ventricular arrhythmias risk if administered alongside medications that lengthen the QT interval on electrocardiogram but has low mineralocorticoid activity; thus, the risk is very low. Therefore, it could be crucial in people receiving azithromycin or hydroxychloroquine, two medications that are advised for COVID-19 therapy, although hydroxychloroquine is not found to have various benefits. A latest clinical trial was done in France in which researchers compared the hydroxychloroquine effect alone with the combined dosage of hydroxychloroquine at 600 mg/day for 10 days with dexamethasone at 20 mg/day for 5 days and 10 mg/day for the remaining 5 days for ARDS patients during COVID-19 at phase III and detected that the patients having combination formulation had 46% death rate and 61.8% death rate when taking hydroxychloroquine only (ClinicalTrials.gov Identifier: NCT04347980). Methylprednisolone or equivalent drug proved to be beneficial in critically ill patients, as per a group of Chinese frontline professionals. They were, however, against the drug’s widespread use, suggesting only for less than 7 days at 0.5–1 mg/kg every day for critically ill COVID-19 patients with pneumonia symptoms (Shang et al., 2020). With live vaccinations, dexamethasone raises the risk of hazardous systemic infection. Based on these latest findings, the WHO was in favor of preliminary results that the dexamethasone should be prescribed as therapeutics for COVID-19 patients and was confirmed to be a lifesaver (WHO Rapid Evidence Appraisal for Covid-19 Therapies (REACT) Working Group, Sterne et al., 2020). As further discoveries emerge, a constant evaluation of the interaction of dexamethasone with other treatments will be required. Earlier clinical uses of methylprednisolone and dexamethasone are summarized in Table 3.

**TABLE 3:** Clinical uses of methylprednisolone and dexamethasone (https://clinicaltrials.gov/).

Drug	Clinical use	Condition	Status
Methylprednisolone	Effects of methylprednisolone on immunological functions and postoperative pain	Methylprednisolone immunological function, postoperative pain	Completed
	The effect of a preoperative single-dose methylprednisolone on the postoperative rehabilitation after abdominal hysterectomy	Hysterectomy, methylprednisolone, postoperative pain	Completed
	Investigation of hospitalization times and mortality according to drug dose in patients given systemic methylprednisolone with a pre-diagnosis of COVID-19 pneumonia; retrospective study	COVID-19, methylprednisolone, pneumonia	Completed
	Effect of methylprednisolone for hantavirus cardiopulmonary syndrome	Hantavirus infections	Completed
Dexamethasone	Dexamethasone induced hiccup in chemotherapy patients treated with methylprednisolone rotation	Dexamethasone induced hiccup in chemotherapy	Completed
	Effect of dexamethasone on labor induction perioperative	Dexamethasone, pregnancy	Completed
	Dexamethasone on perioperative outcome in IBD	Inflammatory bowel diseases, dexamethasone, postoperative complications	Completed
	Dexamethasone in prevention of respiratory morbidity in elective cesarean section in term fetus	Transient tachypnea of the newborn	Completed

*IBD, inflammatory bowel disease.*

## Safety Aspects

Dexamethasone’s safety characteristic is very well known, as it is extensively reported in the literature. A meta-analysis provides evidence that when a short-term high dose is taken, corticosteroids are not responsible for any adverse event that occurred across organ systems (Fernandes et al., 2019). Dexamethasone, the active agent in the pharmaceutical product, has already been well-established for medicinal usage for the last 10 years, with known efficiency and an approved safety concentration. Moreover, even though corticosteroids are usually regarded as safe and extensively used in critically sick patients, the therapeutic threshold while using a higher concentration of corticosteroids in COVID-19 may vary from thresholds for bringing new, costly, and invasive therapies (Young et al., 2020). Methylprednisolone is safe to use orally and parenterally, so methylprednisolone (Medrol) tablets containing doses at 2, 4, 8, 16, and 32 mg are suggested for oral administration. Even intramuscularly given methylprednisolone acetate (Depo-Medrol) and methylprednisolone succinate (Solu-Medrol) are also acceptable.

## Contraindications

Dexamethasone tablets are not advised for patients allergic to any of the ingredients in this drug. The administration of live or live-attenuated vaccines is contradicted for 3 months if patients are already on any immunosuppressive corticosteroids. Methylprednisolone acetate injected suspension is not considered for individuals who are hypersensitive to the drug or its components (Ferrell, 2015). Intramuscular corticosteroid formulations are not recommended for patients with blood disorders like idiopathic thrombocytopenic purpura. Intrathecal delivery of methylprednisolone acetate injection suspension induces severe medical problems. Methylprednisolone acetate injectable suspension is prohibited in systemic mycosis, while for localized joint disorders, an intra-articular route is recommended. In peptic ulcers, heart problems, or hypertension accompanied by heart failure, including varicella and tuberculosis psychoses, diabetes, osteoporosis, or glaucoma, methylprednisolone is being taken with extreme cautiousness (Tseng et al., 2015).

## Conclusion

Severe pneumonia, as well as ARDS in chronic COVID-19 instances, demand corticosteroids in the terminal phases of the infection to lessen inflammation and tissue injury. Limited data are supporting the use of corticosteroids, especially dexamethasone for SARS-CoV-2 infection. Certain studies also indicate that dosages of dexamethasone could reduce death among patients experiencing a severe manifestation of the infection. It is, not, however, prescribed for individuals who still have low symptoms or viral load. More randomized clinical trials using dexamethasone are required to acquire a better knowledge of its involvement in SARS-CoV-2 infection. An intravenous dose of 2 mg/kg/day of methylprednisolone and 6 mg/day of dexamethasone shows similarity in effectiveness by shortening hospital stay or mechanical ventilation and shows better clinical status within 5–10 days in SARS-CoV-2-infected hospitalized patients with pneumonia. The RECOVERY trial had revealed robust demand for dexamethasone, which tends to result in widespread shortages. The findings of this trial have the ability to lessen this desire by delivering methylprednisolone as an optional drug to reduce death among mechanically ventilated COVID-19 sufferers. When taken in line with documented standards, both dexamethasone and methylprednisolone proved to be potent and safe.

## Author Contributions

JM and RR: conceptualization. JM: software. JM, RR, BM, NK, EHC, and NKK: formal analysis. JM, NK, EHC, and NKK: writing—original draft preparation. EHC and NKK: writing—review and editing. NKK: supervision. All authors have read and agreed to the published version of the manuscript.

## Conflict of Interest

The authors declare that the research was conducted in the absence of any commercial or financial relationships that could be construed as a potential conflict of interest.

## Publisher’s Note

All claims expressed in this article are solely those of the authors and do not necessarily represent those of their affiliated organizations, or those of the publisher, the editors and the reviewers. Any product that may be evaluated in this article, or claim that may be made by its manufacturer, is not guaranteed or endorsed by the publisher.

## Footnotes

1
https://www.accessdata.fda.gov/scripts/cder/daf/index.cfm?event=overview.process&ApplNo=011153

2
www.covid19treatmentguidelines.nih.gov

3
https://go.drugbank.com/drugs/DB00741

4
https://clinicaltrials.gov/ct2/show/record/NCT04935515?term=dexamethasone&cond=covid+-19&draw=2&rank=1
